# QSAR study on the removal efficiency of organic pollutants in supercritical water based on degradation temperature

**DOI:** 10.1186/s13065-018-0380-y

**Published:** 2018-02-13

**Authors:** Ai Jiang, Zhiwen Cheng, Zhemin Shen, Weimin Guo

**Affiliations:** School of Environmental Science and Engineering, Shanghai Jiao Tong University, 800 Dongchuan Road, Shanghai, 200240 China

**Keywords:** SCWO process, Organic pollutants, QSAR, Quantum parameters, Fukui indices

## Abstract

This paper aims to study temperature-dependent quantitative structure activity relationship (QSAR) models of supercritical water oxidation (SCWO) process which were developed based on Arrhenius equation between oxidation reaction rate and temperature. Through exploring SCWO process, each kinetic rate constant was studied for 21 organic substances, including azo dyes, heterocyclic compounds and ionic compounds. We propose the concept of T_R95_, which is defined as the temperature at removal ratio of 95%, it is a key indicator to evaluate compounds’ complete oxidation. By using Gaussian 09 and Material Studio 7.0, quantum chemical parameters were conducted for each organic compound. The optimum model is T_R95_ = 654.775 + 1761.910f(+)_n_ − 177.211qH with squared regression coefficient R^2^ = 0.620 and standard error SE = 35.1. Nearly all the compounds could obtain accurate predictions of their degradation rate. Effective QSAR model exactly reveals three determinant factors, which are directly related to degradation rules. Specifically, the lowest f(+) value of main-chain atoms (f(+)_n_) indicates the degree of affinity for nucleophilic attack. qH shows the ease or complexity of valence-bond breakage of organic molecules. BO_x_ refers to the stability of a bond. Coincidentally, the degradation mechanism could reasonably be illustrated from each perspective, providing a deeper insight of universal and propagable oxidation rules. Besides, the satisfactory results of internal and external validations suggest the stability, reliability and predictive ability of optimum model.
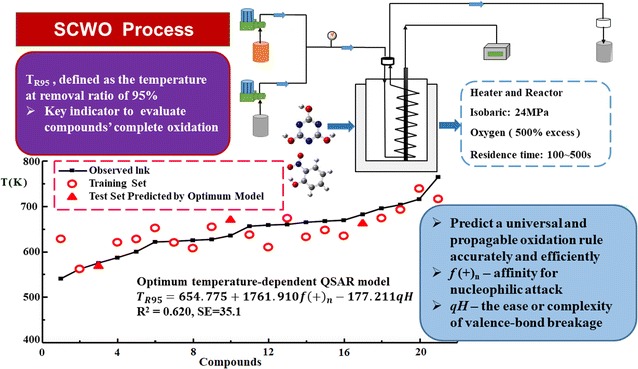

## Introduction

Along with sustainable development of industry, a variety of organic pollutants are released into the environment through different ways, which is potentially noxious to human health and the environment [1, 2]. Due to the complexity of pollutants and the difficulty of destruction, conventional treatments could hardly remove organic compounds. Advanced oxidation processes (AOPs) have been proven particularly effective and fast for treating a wide variety of organic wastewater [3–6]. Supercritical water oxidation (SCWO), one of the AOPs, has been taken as an effective method to degrade substances for higher efficiency, faster reaction rate and less selectivity [7, 8].

Quantitative structure activity relationship (QSAR) models are rapid and cost-effective alternatives to predict theoretical data through building the relationship between molecular structure and physicochemical properties [9, 10]. Several researchers have applied QSAR models to evaluate the eco-toxicity of chemicals without experimental testing [11–13]. At present, numbers of studies have investigated the removal of organic pollutants in SCWO system, which mainly focused on two fields. One is the industrial application of the SCWO technology [14, 15]. Another is exploring relationship between reaction conditions and the degradation efficiency [16, 17]. Compared with factors like pressure and residence time, temperature has been deemed to play a controlling role as reported by Crain et al. [18]. More importantly, the type of treated pollutant accounts for certain appropriate temperature, which is a key indicator when designing and running SCWO system. However, there are seldom researches about theoretical model to offer rapid predictions of systematic effective temperature, which overcome limitations in repeated experiments, like high operational cost and expensive materials [8, 19, 20]. Therefore, in consideration of the rigorous requirements for reaction system, it is of great value and necessity to explore a convenient and efficient QSAR study. This model is significant in both industrial application and theoretical prediction.

It is our emphasis to figure out a common rule available for SCWO system. Also, the impact of Fukui indices and effective temperature on oxidation process were prioritized in QSAR analysis. Primarily, kinetic experiments of diverse compounds were explored. Later, temperature-dependent QSAR models were developed using multiple linear regression. Finally, validations were performed to testify that the optimal model can robustly make predictions.

## Materials and methods

### Reaction system

**Fig. 1:**
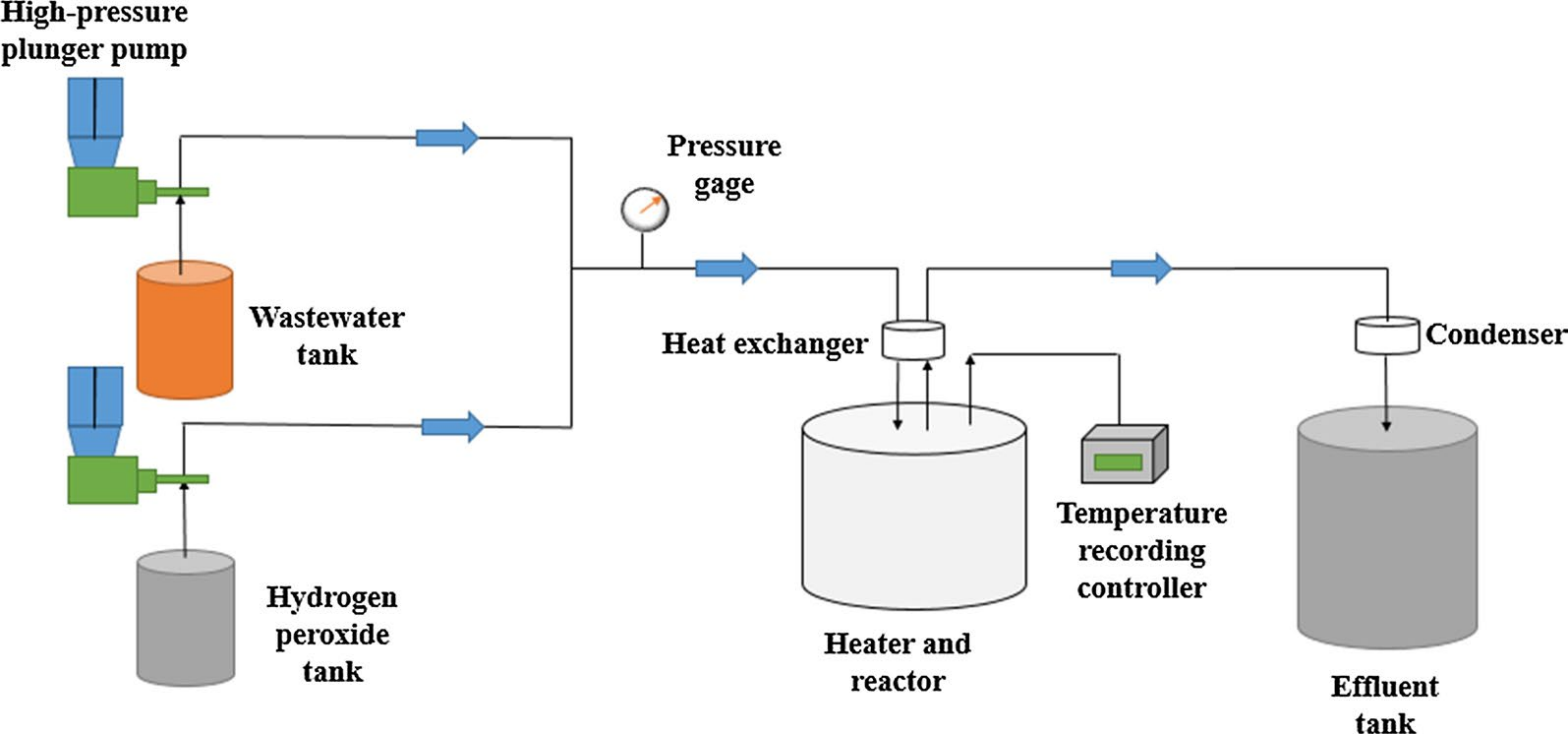
Supercritical flow reactor (SFR) system

The experiments were conducted in a supercritical flow reactor (SFR) system that had been used for previous studies in our laboratory [21]. The major parts consisted of high-pressure plunger pump, hydrogen peroxide tank, waste water tank, gas release valve, check valve, thermometer, pressure gage, heat exchanger, heater and reactor, temperature recording controller, condenser, back pressure regulator and effluent tank. The construction of the SFR was displayed in Fig. 1. It was designed to work under 773.15 K of operating temperature and 30 MPa of operating pressure.

With the aim to study the influence of temperature, compounds thermolysis and oxidation experiments were all performed under isoconcentration (1 g L^−1^) and isobaric (24 MPa) conditions. Meanwhile, reaction system was supplied with sufficient residence time (100–150 s) and oxygen (500% excess). The content of total organic carbon (TOC) in the samples was monitored using a TOC analyzer (TOC-V_CPN_, Shimadzu Corporation, Japan). Hydrogen peroxide (30 wt%) was used as the oxidant in the SCWO experiments and all reagents were analytical pure.

### Arrhenius equation in SCWO system

Temperature is particularly vital in the supercritical reaction conditions. Some orthogonal experiment researches have confirmed the significance of temperature on destruction of the organic structures. The Arrhenius equation is a simple and remarkably accurate formula for the temperature dependence of the reaction rate constant, which can be expressed as follows.

1 $$ k = Aexp^{{\frac{{ - E_{a} }}{RT}}} $$

Based on Eq. (1), an Arrhenius-type Eq. (2) is presented as follows.

2 $$ {\text{T}} = \frac{{E_{a} }}{R(lnA - lnk)} $$

where *A* is the pre-exponential factor and *R* is the gas constant. The units of *A* are identical to those of the rate constant *k* and will vary depending on the order of the reaction. It can be seen that either increasing the temperature T or decreasing the activation energy *E*_*a*_ (for example through the use of catalysts) will result in an increase in rate of reaction. When oxygen exceeds, the degradation process of SCWO system is in accordance with the pseudo-first-order kinetic reaction equation.

3 $$ {\text{T}} = f\left( {\mu ,{\text{ q}}\left( {\text{CN}} \right),{\text{ BO}},{\text{ f}}\left( + \right) \ldots } \right) $$

In short, the Arrhenius equation gives a reliable and applicable principle between ln*k* of oxidation reactions and T (in absolute temperature). Based on present researches focused on the relationship between ln*k* and quantum molecular parameters, function could be assumed as Eq. (3) [22, 23]. It is reasonable to develop a temperatures-dependent QSAR in order to predict oxidation efficiency by theoretical descriptors.

### Computation details

All the calculations were carried out by using chemical density functional theory (DFT) methods in Gaussian 09 (B3LYP/6-311G level) and Material Studio 7.0 (Dmol3/GGA-BLYP/DNP(3.5) basis) [24]. Structure optimization and the total energy calculations of the optimized geometries were based on B3LYP method. During the calculation process, exchange and correlation terms were considered with a B3LYP function (6-311G basis set). Meanwhile, natural population analysis (NPA) of atomic charge was obtained by the same method. The localized double numerical basis sets with polarization functional (DNP) from the DMol3 software were adopted to expand the Kohn–Sham orbitals. The self-consistent field procedure was carried out with a convergence criterion of 10^−6^ a.u. on energy and electron density. Density mixing was set at 0.2 charge and 0.5 spin. The smearing of electronic occupations was set as 0.005 Ha. Molecular parameters of each organic compound are listed in Table 1. They included energy of molecular orbital (E_LOMO_/E_HOMO_), bond order (BO), Fukui indices [f(+), f(−) and f(0)] and so on. In “Optimization” section, they were introduced in detail.

**Table 1:** Molecular descriptors of 21 nitrogenous organic pollutants

Molecule	μ	qH	q (CN)_n_	q (CN)_x_	E_LUMO_	E_HOMO_	BO_n_	BO_x_	f(+)_x_	f(+)_n_	f(−)_n_	f(−)_x_	f(0)_n_	f(0)_x_
	(Debye)	(e)	(e)	(e)	(eV)	(eV)	–	–	(e)	(e)	(e)	(e)	(e)	(e)
Methylene blue trihydrate	12.083	0.239	− 0.366	0.261	− 0.127	− 0.173	1.038	1.418	0.037	0.009	0.037	0.010	0.036	0.012
Rhodamine B	8.788	0.482	− 0.581	0.442	− 0.098	− 0.155	0.924	1.501	0.054	− 0.004	0.055	− 0.004	0.055	− 0.004
Eriochrome blue black R	7.110	0.497	− 0.271	0.451	− 0.009	− 0.276	1.187	1.532	0.046	0.001	0.039	0.005	0.046	0.007
*o*-Nitroaniline	4.726	0.421	− 0.254	0.212	− 0.087	− 0.230	1.199	1.462	0.082	0.023	0.115	0.048	0.067	0.044
Isatin	4.622	0.409	− 0.254	0.220	− 0.105	− 0.249	0.878	1.392	0.119	0.026	0.076	0.017	0.096	0.025
3,4-Dichloroaniline	5.034	0.381	− 0.774	0.202	− 0.027	− 0.215	1.118	1.424	0.108	0.037	0.139	0.039	0.091	0.050
*N*,*N*-dimethylbenzylamine	0.646	0.213	− 0.503	− 0.031	− 0.009	− 0.220	0.973	1.397	0.105	0.002	0.244	− 0.016	0.123	0.026
2-Nitrophenol	3.579	0.492	− 0.251	0.370	− 0.107	− 0.258	0.983	1.438	0.115	0.023	0.125	0.025	0.089	0.037
Nitrobenzene	4.541	0.238	− 0.191	0.060	− 0.097	− 0.288	1.323	1.390	0.123	0.024	0.074	− 0.001	0.087	0.025
Aniline	1.715	0.362	− 0.783	0.192	0.001	− 0.198	1.288	1.414	0.123	0.045	0.164	0.062	0.105	0.057
Methyl orange	8.801	0.217	− 0.547	0.252	− 0.009	− 0.284	0.975	1.582	0.094	0.012	0.032	0.016	0.086	0.016
Crystal violet	14.763	0.271	− 0.424	0.260	− 0.101	− 0.151	0.928	1.488	0.053	0.002	0.051	0.004	0.052	0.007
Phenol	1.344	0.460	− 0.291	0.342	− 0.012	− 0.229	1.320	1.396	0.124	0.057	0.136	0.074	0.104	0.073
5-Chloro-2-methylbenzylamine	3.827	0.383	− 0.782	0.215	− 0.010	− 0.208	1.002	1.378	0.112	0.026	0.141	0.021	0.092	0.024
*p*-Dimethylaminobenzaldehyde	6.427	0.218	− 0.420	0.414	− 0.047	− 0.210	0.956	1.459	0.141	0.018	0.100	0.027	0.098	0.022
Indole	2.201	0.399	− 0.543	0.168	− 0.208	− 0.015	1.093	1.563	0.112	0.029	0.121	0.030	0.107	0.037
1,10-Phenanthroline monohydrate	3.198	0.207	− 0.422	0.192	− 0.061	− 0.238	1.101	1.570	0.063	0.025	0.134	0.018	0.093	0.023
Sulfanilic acid	5.869	0.487	− 0.760	0.215	− 0.038	− 0.237	1.087	1.436	0.084	0.060	0.087	0.048	0.073	0.061
1-Methylimidazole	4.131	0.203	− 0.492	0.202	− 0.230	0.019	0.909	1.597	0.165	0.042	0.176	0.026	0.161	0.034
Cyanuric acid	3.096	0.489	− 0.787	0.954	0.141	− 0.421	1.127	1.425	0.109	0.097	0.210	0.060	0.154	0.082
Melamine	0.000	0.389	− 0.765	0.642	0.023	− 0.232	1.179	1.376	0.092	0.074	0.107	0.044	0.095	0.068

In order to obtain optimum number of variables for the correlation model, stepwise regression procedure was used to build QSAR models by the SPSS 17.0 for windows program. The quality of derived QSAR was evaluated in accordance with the squared regression coefficient (R^2^), the standard error (SE) as well as *t* test and the Fisher test. The internal validation was performed by leave-one-out cross-validation (q^2^), and the external validation was also computed (Q_EXT_^2^). In both validation methods, a validation value greater than 0.5 indicates a robust and predictive model.

## Results and discussion

**Fig. 2:**
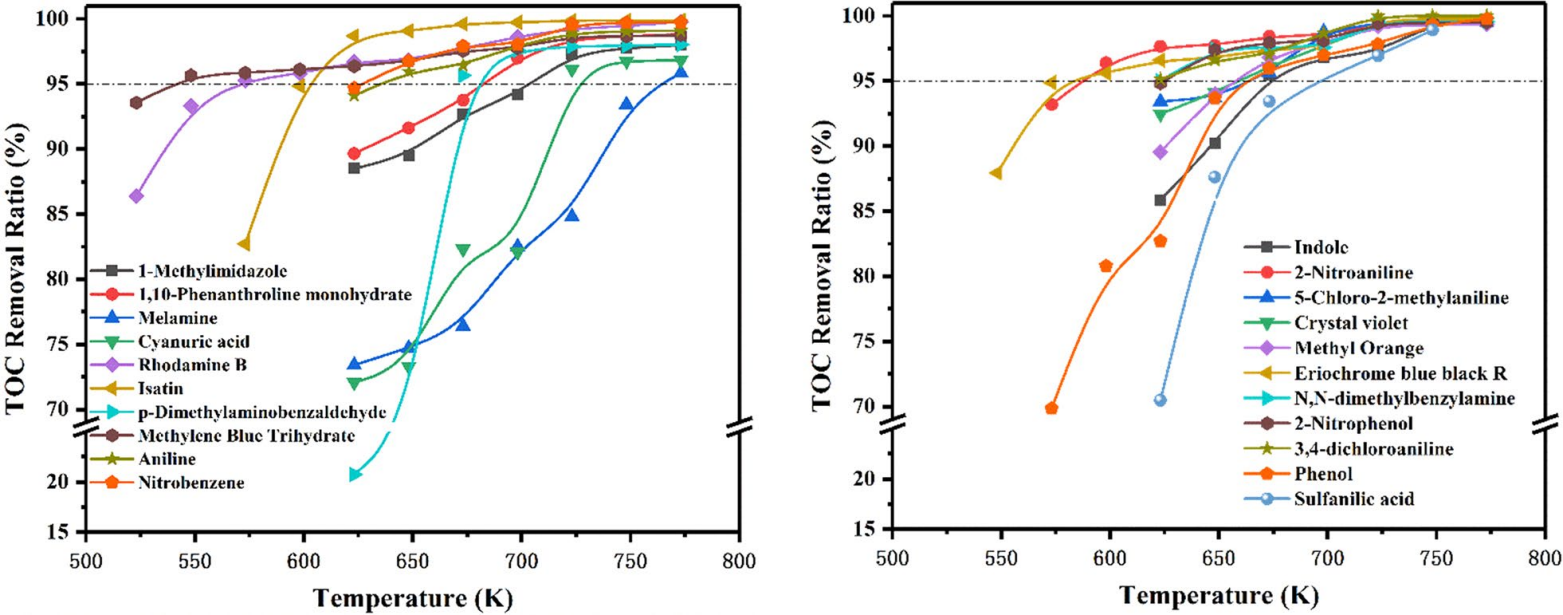
TOC removal of 21 organic pollutants in SCWO system at different temperatures

The degradation process of 21 kinds of organic pollutants was investigated at 24 Mpa from the subcritical to supercritical temperature with 500% excess oxygen. Sampling occurred from 523.15 to 773.15 K. An important design consideration in the development of SCWO is the optimization of operating temperature. As shown in Fig. 2, TOC degradation efficiency of compounds tends to be higher with the increase of operating temperature. When the temperature reached 773.15 K, most organics could be totally oxidized into water and carbon dioxide. The compounds are considered to be completely removed while the degradation efficiency reaches 95%. Consequently, we propose the concept of T_R95_, which is defined as the temperature at removal ratio of 95%, as the key indicator to evaluate compounds’ complete oxidation. T_R95_ values of the reaction system are distinguished, ranging from 540.65 K (of Methylene blue trihydrate) to 764.26 K (of melamine), which indicate that organic compounds in this study are different and complex. Thus, among diverse molecules, it is significant to set up a temperature-dependent QSAR which can predict SCWO thermodynamics and oxidization activities and conclude universal rules.

### Optimization

The structure optimization of organic matter and the calculation of the total energy for the optimized geometry are based on the B3LYP method in Gaussian 09 and Dmol3 code in Material Studio 7.0. All quantum descriptors are directly available from the output file of two software. Finally, as shown in Table 1, we got the following 15 molecular descriptors of organics: dipole moment (μ), most positive partial charge on a hydrogen atom (qH), most negative or positive partial charge on a carbon or nitrogen atom (q(CN)_n_/q(CN)_x_), energy of the lowest unoccupied molecular orbital (E_LUMO_), energy of highest occupied molecular orbital (E_HOMO_), minimum or maximum of bond order values in the molecule (BO_n_/BO_x_), and maximum or minimum of Fukui indices [f(+)_x_/f(+)_n_, f(−)_x_/f(−)_n_ and f(0)_x_/f(0)_n_].

### Main theoretical parameters

All organic pollutants and their 14 respective molecular parameters are listed in Table 1. These theoretical parameters are important to observe which sites are active to be attacked and which bonds are sensitive to be ruptured. Fukui indices, frontier molecular orbits, bond orders are key concepts to portray the decomposition sequence of organic structure in oxidation.

Fukui indices are defined as affinity for radical attack. They are significant for analysis of site reactive selectivity among the oxidation paths, as hydrogen substitution by oxidant radicals and addition of oxidant group to double bonds are the most events. In this study, f(+)_n_, f(−)_n_ and f(0)_n_ stand for the minimum values of nucleophilic attack, electrophilic attack and ^·^OH radical attack respectively. f(+)_x_, f(−)_x_ and f(0)_x_ do for their respective maximum values on main chain of both carbon and nitrogen atoms. The average level of f(+)_n_, f(−)_n_ and f(0)_n_ are 0.030e, 0.026e, and 0.035e respectively, while those of f(+)_x_, f(−)_x_ and f(0)_x_ are 0.098e, 0.113e and 0.091e, respectively. The variation of each Fukui indices was extremely huge. Moreover, it is noticeable that cyanuric acid and 1-methylimidazole always have high values of all Fukui indices.

As stated earlier, NPA has been developed to calculate atomic charges and orbital populations of molecular wave functions in general atomic orbital basis sets. NPA is an alternative to conventional Mulliken population analysis. It improves numerical stability and describes the charge distribution better. qH is considered as charge of hydrogen atoms in the molecular structure system. q(CN)_n_ and q(CN)_x_, refer to the minimum and maximum of most negative partial charge on a main-chain carbon or nitrogen atom in the molecule. In this study, qH, q(CN)_n_ and q(CN)_x_ have the average values of 0.355e, − 0.498e and 0.295e respectively. At the same time, the maximum of qH, q(CN)_n_ and q(CN)_x_ reach 0.497e, − 0.191e and 0.945e respectively, while the minimum of them are 0.203e, − 0.787e and − 0.032e respectively. It is also noticeable that the distinguish between the largest and the smallest value of q(CN)_x_ is 0.977e, which is a wide range for compounds, leading the challenges and values of our study.

### Construction of QSAR models

Using the obtained molecular descriptors as variables, the correlation models of the predictable rate constants were developed by Multivariate linear regression (MLR) method. There are three out of 14 descriptors, f(+)_n_, qH, and BO_x_, correlated well with T_R95_ respectively. With the exclusion of parameters of the least importance, the relationship for degradation rate of organic pollutants was established using MLR analysis. Three effective models with their associated data indices are shown in Table 2. All the predictable values of T_R95_ values (Pred.) by three QSAR models and the experimental values are listed in Table 3.

**Table 2:** Regression models for calculating T_R95_ of organic pollutants

No	Model	R^2^	SE	F	q^2^	Q_EXT_^2^
1	T_R95_ = 599.849 + 1492.671f(+)_n_	0.502	39.127	19.121	0.380	0.365
2	T_R95_ = 654.775 + 1761.910f(+)_n_ − 77.211qH	0.620	35.087	14.702	0.570	0.741
3	T_R95_ = 396.855 + 1874.189f(+)_n_ − 158.091qH + 169.801BO_x_	0.665	33.905	11.255	0.468	0.884

**Table 3:** Tested and three predicted T_R95_ values of 21 organic pollutants

No	Molecule	Tested (K)	Pred. (K)		
			1	2	3
1	Methylene blue trihydrate	540.653	613.283	628.263	616.633
2	Rhodamine B	562.093	593.883	562.323	568.053
3^a^	Eriochrome blue black R	575.303	601.343	568.463	580.313
4	*o*-Nitroaniline	587.053	634.183	620.653	621.713
5	Isatin	600.023	638.663	628.063	617.203
6	3,4-Dichloroaniline	621.533	655.083	652.393	647.683
7	*N*,*N*-dimethylbenzylamine	622.873	602.833	620.553	604.143
8	2-Nitrophenol	625.273	634.183	608.073	606.393
9	Nitrobenzene	627.043	635.673	654.843	640.203
10^a^	Aniline	635.453	667.023	669.833	664.133
11	Methyl orange	656.223	617.763	637.443	653.653
12	Crystal violet	658.803	602.833	610.273	610.363
13	Phenol	659.973	684.933	673.593	667.993
14	5-Chloro-2-methylbenzylamine	664.803	638.663	632.673	619.043
15	*p*-Dimethylaminobenzaldehyde	667.433	626.723	647.833	643.903
16	Indole	669.283	643.143	635.113	653.493
17^a^	1,10-Phenanthroline monohydrate	682.103	637.173	662.103	677.503
18	Sulfanilic acid	695.473	689.413	674.093	676.153
19	1-Methylimidazole	703.193	662.543	692.733	714.683
20	Cyanuric acid	715.433	744.643	738.863	743.383
21	Melamine	764.263	710.313	716.103	707.663

^a^ Samples in an external test set

**Fig. 3:**
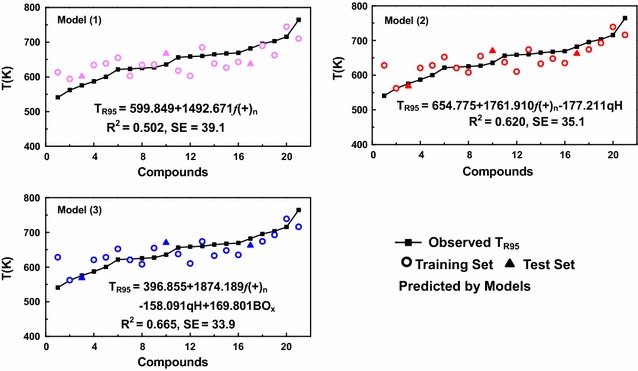
Three QSAR models for degradation rules of organic pollutants

It is widely reported that favorable models are generally determined by R^2^ and SE [25, 26]. According to the predictable performance shown in Fig. 3 [model (1), (2) and (3)], R^2^ increase with the number of variables. To avoid the over-parameterization of model, the value of leave-one-out cross-validation q^2^ closer to corresponding R^2^ was chosen as the breakpoint criterion. Therefore, model (2) with two descriptors was considered as the best one, which also fits well with both ideal regression (R^2^ = 0.620 > 0.600) and internal validation (q^2^ = 0.570 > 0.500). These statistics guarantee that the model is very robust and predictive. Apart from that, it can be seen from Fig. 3 that model (2) also had the best fitting curve between the predicted and experimental data. Tested T_R95_ values increase almost linearly with all organic pollutants except for methylene blue trihydrate and crystal violet. Most T_R95_ values predicted by optimum model are evenly distributed around regression line. The measured T_R95_ and those calculated with model (2) are in observed to be in good agreement. In this view, it is worthwhile and reasonable to predict degradation rules by model (2).

Model (2), the optimum model, contains two variables f(+)_n_ and qH. Each variable plays an important role in the supercritical water oxidation process, revealing the reaction rules. Firstly, f(+)_n_ is a measurement of the affinity for nucleophilic attack. When f(+)_n_ is larger, it is easier of main-chain atom (carbon or nitrogen) to be attacked. So, compounds with high f(+)_n_ values have weak endurance to oxidants and not so high appropriate temperature, such as isatin and 3,4-dichloroaniline. Secondly, qH shows the non-uniformity of electric charge on hydrogen, which indicates the ease or complexity of valence-bond breakage of organic molecules. Take Eriochrome blue black R for example, it is tested as high qH value (0.497e), leading to its low efficient degradation temperature (T_R95_ = 575.30 K).

### Validation and performance

To check the stability of optimum model, leave-one-out cross-validation, pairwise correlation coefficients, *t* test and Fisher test are employed using SPSS 17.0 for window program. The values of leave-one-out cross-validation q^2^ of three models are shown in Table 2. As can be seen from that, q^2^ of model (2) is the best of three models and is larger than 0.500. Pairwise correlation coefficients of model (2) are shown in Table 4. The correlation coefficients order between the tested values of T_R95_ and independent variables are as follows: f(+)_n_ > qH > BO_x_. The correlation coefficient is 0.346 between f(+)_n_ and qH, so model (2) is acceptable.

**Table 4:** Correlation coefficient(r) matrix for variables of model (2)

	T_R95_	f(+)_n_	qH	BO_x_
T_R95_	1.000	–	–	–
f(+)_n_	0.868	1.000	–	–
qH	− 0.096	0.346	1.000	–
BO_x_	0.053	− 0.301	− 0.259	1.000

The standard regression coefficients and t values of independent variables for model (2) are listed in Table 5. And all the absolute t values are larger than the standard one, suggesting that four variables are able to accept. Furthermore, we could evaluate the correlation degree of each independent variable by calculating their variation inflation factors (VIF). VIF = 1/(1 − r^2^), in which r is the correlation coefficient of multiple regressions between one variable and the others. If VIF ranges from 1.000 to 5.000, the related equation is acceptable; and if VIF is larger than 10.000, the regression equation is unstable and recheck is necessary. It can be seen from Table 5, most VIF values are slightly over 1.000 and the maximum is 5.226, indicating model (2) has obvious statistical significance. An external validation of suggested model has been performed for three compounds, which are not involved in the model-building process. A test set was randomly selected with interval of seven, including Eriochrome blue black R, aniline and 1,10-phenanthroline monohydrate. The Q_EXT_^2^ value (as shown in Table 2) of 0.741 (> 0.500) indicates that suggested models have good predictive potential.

**Table 5:** Checking statistical values for three models

	Regression coefficients	t	Sig.	VIF
Model (1)				
Constant	599.849	24.549	0.000	–
f(+)_n_	1492.671 ± 0.708	4.373	0.000	4.055
Model (2)				
Constant	654.775	14.650	0.000	–
f(+)_n_	1760.252 ± 0.835	5.396	0.000	5.226
qH	− 177.214 ± 0.376	− 2.372	0.029	1.010
Model (3)				
Constant	396.855	0.716	0.035	–
f(+)_n_	1874.189 ± 0.889	5.782	0.000	4.067
qH	− 158.091 ± 0.328	− 2.157	0.046	1.009
BO_x_	169.801 ± 0.225	1.509	0.150	1.003

## Conclusions

Appropriate reaction temperature is an important factor to design and operate the supercritical water oxidation (SCWO) system. In this paper, QSAR models for organic compounds were developed on the basis of Arrhenius equation between oxidation reaction rate and temperature in SCWO process. According to the calculations of molecular parameters by DFT methods in Gaussian 09 and Material Studio 7.0, f(+)_n_, qH and BO_x_ appeared in established QSAR models focusing on the impact of Fukui indices and effective temperature, which reveals they are significant in understanding degradation mechanism. The optimum model has ideal regression and internal validation (R^2^ = 0.620, SE = 35.1). The results of *t* test and Fisher test suggested that the model exhibited optimum stability. Both internal and external validations showed its robustness and predictive capacity. Coincidentally, the obtained determinant factors are included with degradation process including the affinity for attack, difficulty of electron loss as well as non-uniformity of valence bond. Together with them, the degradation mechanism could reasonably be illustrated from each perspective, providing a deeper insight of universal and propagable oxidation rules.
